# Supplementary material for: First-Line Chemoimmunotherapy versus Sequential Platinum-Based Chemotherapy Followed by Immunotherapy in Patients with Non-Small Cell Lung Cancer with ≤49% Programmed Death-Ligand 1 Expression: A Real-World Multicenter Retrospective Study
Source: Cancers (Basel). 2023 Oct 14;15(20):4988. doi: 10.3390/cancers15204988 (PMC10605814; doi:10.3390/cancers15204988)
Supplement: Supplementary file 1 [file cancers-15-04988-s001.zip › cancers-2653647-supplementary.pdf]

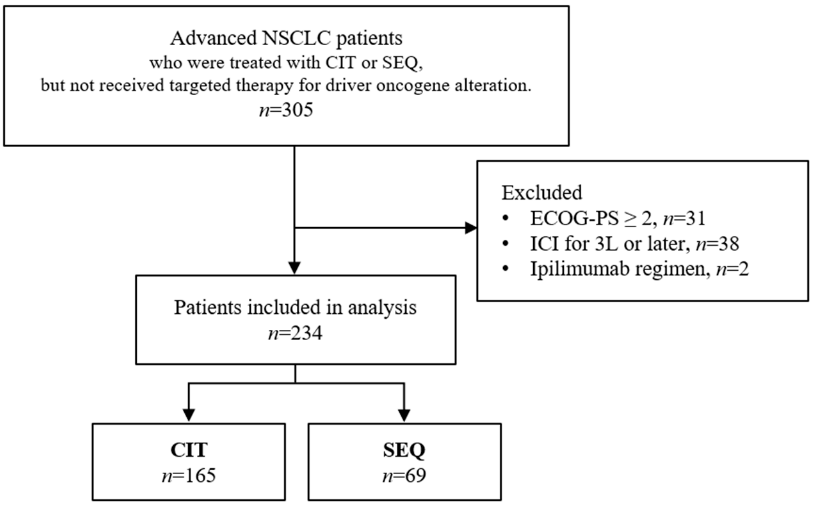


**Supplemental Figure S1. Flow-diagram of patients included in this study.**

CIT, chemoimmunotherapy; ECOG-PS, Eastern Cooperative Oncology Group performance status; 1L, first-line; NSCLC, non-small cell lung cancer; 2L, second-line; SEQ, sequential treatment with platinum-based chemotherapy followed by immune checkpoint inhibitors; 3L, third-line.

**Supplemental Table S1. Characteristics of patients enrolled in the SEQ and CIT groups in the propensity score-matched cohort.**

| | | **PSM cohort** | | |
|---|---|---|---|---|
| | Group –*n*. (%) | CIT (*n*=69) | SEQ (*n*=69) | *p*-value |
| Age -y, median (range) | | 71 (45, 85) | 70 (33, 82) | 0.700 |
| Sex -n. (%) | Male | 62 (89.9) | 63 (91.3) | 1.000 |
| | Female | 7 (10.1) | 6 (8.7) | |
| Smoking status -n. (%) | Never | 3 (4.3) | 6 (8.7) | 0.493 |
| | Current or former | 66 (95.7) | 63 (91.3) | |
| Histology -n. (%) | Sq. | 30 (43.5) | 30 (43.5) | 1.000 |
| | Non-Sq. | 39 (56.5) | 39 (56.5) | |
| PD-L1 expression -n. (%) | 1-49% | 56 (81.2) | 55 (79.7) | 1.000 |
| | <1% | 13 (18.8) | 14 (20.3) | |

| | | | | |
|---|---|---|---|---|
| Stage -n. (%) | IIIB or IIIC | 7 (10.1) | 6 (8.7) | 1.000 |
| | Recurrence or IV | 62 (89.9) | 63 (91.3) | |

CIT, chemoimmunotherapy; Sq., squamous cell carcinoma; PD-L1, programmed death-ligand 1; PSM, propensity score-matched; SEQ, sequential treatment with platinum-based chemotherapy followed by immune checkpoint inhibitors.

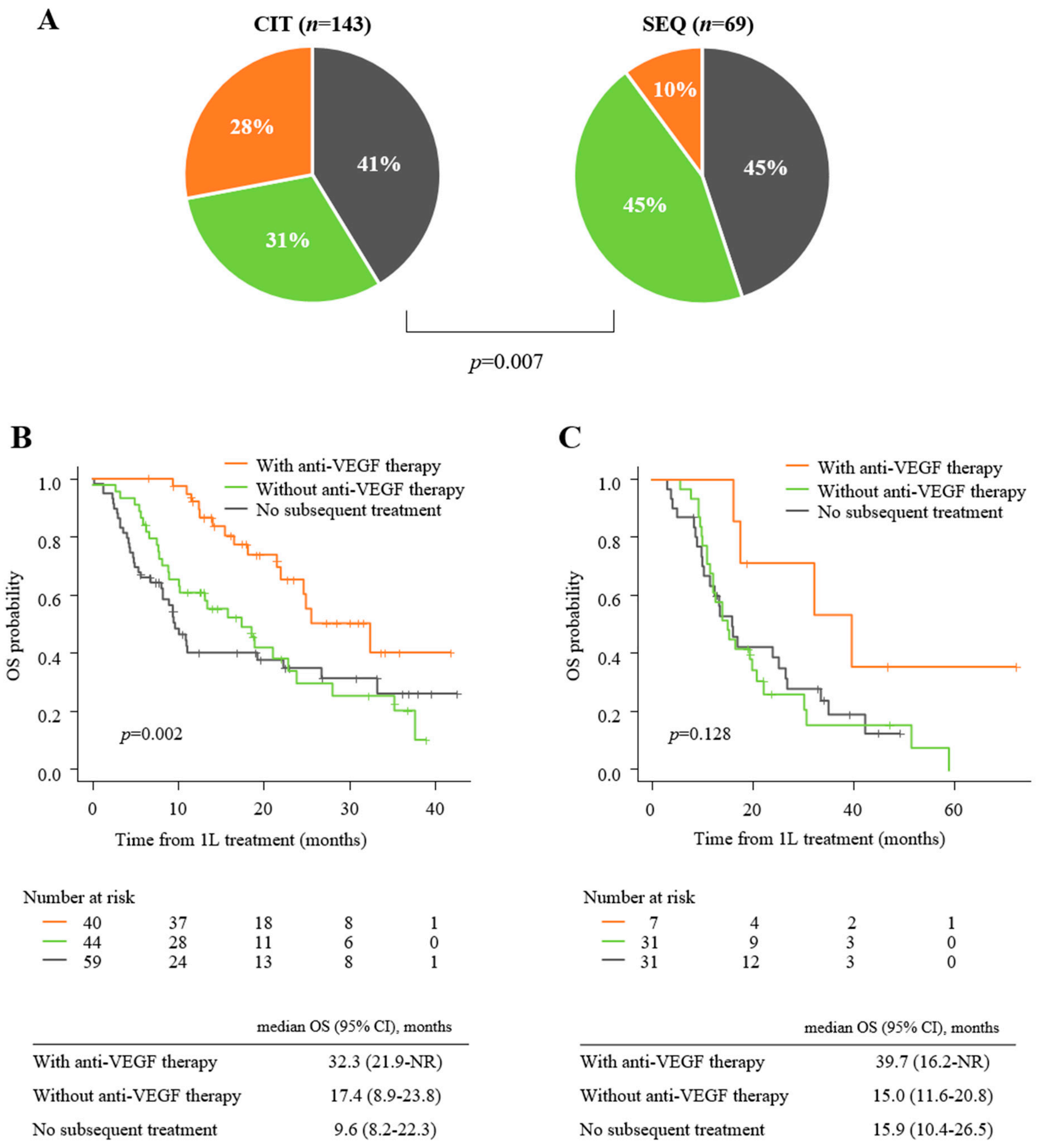


**Supplemental Figure S2. Transition to subsequent chemotherapy after CIT or SEQ and overall survival.**

A total of 143 patients in the CIT group and 69 in the SEQ group who terminated immune checkpoint inhibitor (ICI) treatment were selected for analysis. **(A)** Transition rate to subsequent chemotherapy after ICI treatment. Fisher's exact test was used to compare the differences in subsequent treatment options between the CIT and SEQ groups. **(B, C)** OS in patients treated with (**B**) CIT or (**C**) SEQ according to subsequent chemotherapy: chemotherapy with anti-vascular endothelial growth factor (VEGF) therapy vs. chemotherapy without anti-VEGF therapy vs. no treatment. *P* values were calculated using log-rank analysis.

CIT, chemoimmunotherapy; SEQ, sequential treatment with platinum-based chemotherapy followed by immune checkpoint inhibitors; OS, overall survival; 1L, first-line; CI, confidence interval; NR, not reached.

**Supplemental Table S2. Adverse events with CTCAE grade 3 or 4**

| | **CIT** (*n*=165) | **SEQ** (*n*=69) | |
|---|---|---|---|
| -*n*. (%) | 1L-CIT | 1L-Chemo | 2L-ICI |
| AE, Grade 3 ≤ | 71 (43) | 22 (31.9) | 10 (14.5) |
| Neutropenia | 39 (23.6) | 14 (20.3) | 0 (0) |
| Anemia | 24 (14.5) | 1 (1.4) | 0 (0) |
| Thrombocytopenia | 11 (6.7) | 5 (7.2) | 0 (0) |
| Interstitial lung disease | 10 (6.1) | 1 (1.4) | 5 (7.2) |
| Hepatobiliary disorders | 6 (3.6) | 0 (0) | 1 (1.4) |
| Febrile neutropenia | 4 (2.4) | 1 (1.4) | 0 (0) |
| Malaise | 1 (0.6) | 1 (1.4) | 1 (1.4) |
| Colitis | 2 (1.2) | 0 (0) | 1 (1.4) |
| Nausea | 1 (0.6) | 0 (0) | 2 (2.9) |
| Infusion related reaction | 2 (1.2) | 0 (0) | 0 (0) |
| Stevens-Johnson syndrome | 0 (0) | 1 (1.4) | 0 (0) |
| Stroke | 0 (0) | 1 (1.4) | 0 (0) |
| Gastric ulcer | 0 (0) | 1 (1.4) | 0 (0) |
| Adrenal insufficiency | 1 (0.6) | 0 (0) | 0 (0) |

| | | | |
|---|---|---|---|
| Rash | 1 (0.6) | 0 (0) | 0 (0) |
| Pneumothorax | 1 (0.6) | 0 (0) | 0 (0) |
| Syncope | 1 (0.6) | 0 (0) | 0 (0) |
| Myalgia | 1 (0.6) | 0 (0) | 0 (0) |
| Uveitis | 1 (0.6) | 0 (0) | 0 (0) |

AE, adverse event; Chemo, chemotherapy; CIT, chemoimmunotherapy; CTCAE, Common Terminology Criteria for Adverse Events; ICI, immune checkpoint inhibitor; SEQ, sequential treatment with platinum-based chemotherapy followed by immune checkpoint inhibitors.
